# *AwAreA* Regulates Morphological Development, Ochratoxin A Production, and Fungal Pathogenicity of Food Spoilage Fungus *Aspergillus westerdijkiae* Revealed by an Efficient Gene Targeting System

**DOI:** 10.3389/fmicb.2022.857726

**Published:** 2022-03-31

**Authors:** Gang Wang, Yujie Li, Bolei Yang, Erfeng Li, Wenqing Wu, Peidong Si, Fuguo Xing

**Affiliations:** ^1^Key Laboratory of Agro-Products Quality and Safety Control in Storage and Transport Process, Ministry of Agriculture and Rural Affairs, Institute of Food Science and Technology, Chinese Academy of Agricultural Sciences, Beijing, China; ^2^Horticulture and Landscape College, Tianjin Agricultural University, Tianjin, China

**Keywords:** *Aspergillus westerdijkiae*, uridine auxotrophy, gene target, ochratoxin A, nitrogen source, *AreA*

## Abstract

*Aspergillus westerdijkiae*, the producer of ochratoxin A (OTA), which is of worldwide concern, is an import fungal species in agriculture, food, and industry. Here, we got the uridine auxotrophic mutant of *A. westerdijkiae* by deleting *AwpyrG*. The Δ*AwpyrG* could be used for bio-transformation with exogenous *AfpyrG* expression cassette as a selection marker. In order to enhance the efficiency of gene targeting, *Awku70* and *Awlig4* were homologously deleted from Δ*AwpyrG*. The efficiencies of homologous replacement for Δ*Awku70* and Δ*Awlig4* were 95.7 and 87.0% in the deletion of *AwAreA*, respectively, demonstrating a drastic increase from 4.3% of the wild type (WT) strain. Furthermore, the function of *AwAreA* was identified with *AwAreA* deletion mutant and the control strain Δ*Awku70*. *AwAreA* regulated the growth and conidiation of *A. westerdijkiae* in response to nitrogen sources. The concentration of OTA for Δ*Awku70* was in the range of 19.4 to 186.9 ng/cm^2^ on all kinds of nitrogen sources. The OTA production influenced by the deletion of *AwAreA* was different based on nitrogen sources. Pathogenicity assays on pears, grapes, salted meat, and cheese showed that *AwAreA* acted as a negative regulator in the infection of food substrates. Therefore, the genetic methods and engineered strains enable us to substantially expand the use of *A. westerdijkiae*, one of more than twenty OTA-producing fungi, in the study of mycotoxin biosynthesis and regulation, and consequently to aim at providing new ways for controlling this pathogen.

## Introduction

Ochratoxin A (OTA) was first characterized in 1965 in South Africa (Van der Merwe et al., 1965b) and listed as a possible carcinogenic substance (group 2B) by the International Agency for Research on Cancer [IARC] (1993). The chemical structure of OTA has been identified in the same year (Van der Merwe et al., 1965a). The biosynthesis of OTA was first studied with the aid of radioactive precursors, demonstrating that 1−^14^C-phenylalanine was incorporated into OTA when it was supplied in the culture of *Aspergillus ochraceus* (Steyn et al., 1970). In 2005, a genomic region encoding a polyketide synthase and a non-ribosomal peptide synthetase encoding gene was cloned and characterized through the construction of a phage lambda genomic gene bank (Karolewiez and Geisen, 2005). Since then, the research on biosynthesis and regulation of OTA had mostly focused on the identification of gene functions of single or multiple genes (Gallo et al., 2012, 2017; Wang et al., 2017, 2019). Until recently, genomic sequence technologies opened a new era in the study of OTA biosynthesis and regulation by significantly increasing the catalog of ochratoxigenic fungal genome sequences (Vesth et al., 2018; Wang et al., 2018; Ferrara et al., 2020; Kjærbølling et al., 2020). Homologous integration enables precise disruption or replacement of genes and provides an effective way to study gene function. Although the function of some genes has been elucidated, the majority of genes related to fungal growth and OTA biosynthesis are still obscure. Moreover, more than twenty species in *Aspergillus* and *Penicillium* were detected to produce OTA (Visagie et al., 2014; Wang Y. et al., 2016). They contaminate different food commodities such as cereals, beans, nuts, fruits, and spices. However, there was no efficient gene targeting system to study neither the biosynthesis and regulation of OTA nor the interaction between fungi and foodstuff. One reason for the lack of knowledge is the low gene targeting frequency of the OTA-producing fungi.

In general, both homologous recombination (HR) and non-homologous end-joining (NHEJ) are important pathways to repair DNA double-strand breaks (Scully et al., 2019). HR is also employed to knockout or express genes at the designated site by introducing foreign DNA segments. *Saccharomyces cerevisiae* shows a high homologous integration rate for the domination of the HR system for DNA repair. In contrast, some organisms like filamentous fungi seem to prefer to repair DNA by NHEJ, resulting in the random integration of exogenous DNA into chromosomes. Therefore, inhibiting or eliminating the NHEJ pathway is a strategy to increase gene targeting frequency. The process of the NHEJ pathway is mediated by the Ku70-Ku80 heterodimer, the DNA ligase IV (Lig4)–Xrcc4 complex, and DNA-dependent protein kinase catalytic subunit (DNA-PKcs) (Ninomiya et al., 2004). The conservative NHEJ through evolution allows people to develop gene targeting systems in fungi with high efficiency, e.g., *Aspergillus nidulans*, *Neurospora crassa*, and *Tolypocladium ophioglossoides* (Ishibashi, 2006; Ding et al., 2019; Chen et al., 2020).

The usability of the selection marker is another important factor for genetic transformation. Until now, the genetic manipulations of all the OTA-producing fungi, such as *A. ochraceus* (Wang et al., 2018), *Aspergillus niger* (Zhang et al., 2018), *Aspergillus carbonarius* (Maor et al., 2021), and *Penicillium verrucosum* (O’Callaghan et al., 2013), have utilized hygromycin B resistance gene as a selection marker for the transformation. Indeed, uridine auxotrophy has been widely exploited as a marker recycling system in some filamentous fungi (Gouka et al., 1995). *pyrG*/*URA3* orthologs are genes that encode orotidine 5′-monophosphate decarboxylases (OMPdecase) or orotidylate decarboxylase that catalyzes the decarboxylation of orotidine 5′-monophosphate to form uridine monophosphate. That is the key step for the biosynthesis of uridine triphosphate pyrimidine nucleotides. *pyrG*/*URA3* can also catalyze 5′-fluoroorotic acid (5-FOA) to the toxic 5′-fluoro-UMP, which inhibits fungal growth. Therefore, a *pyrG*/*URA3* defective strain has often been isolated by positive selection *via* resistance to 5-FOA in the presence of uridine.

Nitrogen is an essential nutrient for fungal growth and the production of mycotoxins in food substrate. Nitrogen availability also plays a critical role in plant–pathogen interactions. That is why different ochratoxigenic fungi adapt to diverse nitrogen derived from food substrates (Koteswara et al., 2012). It was reported that glutamine was the optimal nitrogen source for aflatoxin production in *Aspergillus flavus* when grown in Czapek Dox medium (Wang et al., 2017). Glucose-containing sugars and complex nitrogen sources were favorable conditions for patulin production (Zong et al., 2015). Fungi have evolved sophisticated mechanisms for nitrogen acquisition and utilization (Tang et al., 2020). A mechanism known as nitrogen catabolite repression (NCR) is used to regulate nitrogen utilization. In the presence of the preferred nitrogen sources like ammonium and glutamine, the utilization of less favored nitrogen sources such as nitrate is usually repressed. *AreA*, the GATA-type transcription factor, is known for its involvement in nitrogen metabolism. It has been proved the disruption of *AreA* inhibited the growth of *A. nidulans* on several nitrogen sources (Langdon et al., 1995). *AreA* orthologs also play crucial roles in fungal development, conidia production, and virulence of plant pathogens in genus *Fusarium* (Tang et al., 2020), *Colletotrichum* (Bi et al., 2017), etc. However, the nitrogen dependency and the transcription factor *AreA* of most OTA-producing fungi have not been well understood.

*Aspergillus westerdijkiae* is one of the most notorious ochratoxigenic fungi contaminating cereal, fruit, salted meat, and cheese. Several isolates identified as the original OTA-producing *A. ochraceus* were reclassified as *A. westerdijkiae* (Frisvad, 2004; Gil-Serna et al., 2009, 2020), showing its critical roles in OTA contamination. Here, we established the uridine auxotrophic *A. westerdijkiae* and further developed an efficient gene targeting system by disruption of *Awku70* and *Awlig4* in the NHEJ pathway. As a proof of concept, we deleted the transcription factor *AreA* involved in nitrogen metabolism and explored the morphological development, OTA biosynthesis, and fungal virulence of *A. westerdijkiae* responding to nitrogen sources and modulated by *AwAreA*.

## Materials and Methods

### Fungal Strains

The strain *A. westerdijkiae* Fc-1 originates from the customary *A. ochraceus* Fc-1 (Wang et al., 2018). We realized that the *A. ochraceus* Fc-1 should be classified as *A. westerdijkiae* based on the β-*tubulin* sequence (GenBank accession no. OM283273) and morphological identification. As shown in Supplementary Figure 1, a yellowish-orange pigment was produced on Czapek Yeast Autolyzate agar medium when *A. westerdijkiae* Fc-1 was cultured at 33°C, which was not observed for strain *A. ochraceus* ITEM 7043. This was consistent with the description for the morphological differences in *Aspergillus* section Circumdati (Visagie et al., 2014). Fungal strains used in this study are listed in Table 1.

**TABLE 1:** Strains used in this study.

Strain name	Gene type	Parental strain
Fc-1	WT	
Δ*AwPyrG*	Δ*AwPyrG*	Fc-1
Δ*Awku70*	Δ*AwPyrG*Δ*Awku70*::*AfpyrG*	Δ*AwPyrG*
Δ*Awlig4*	Δ*AwPyrG*Δ*Awlig4*::*AfpyrG*	Δ*AwPyrG*
Δ*AwAreA-WT*	Δ*AwAreA*::*hygR*	Fc-1
Δ*AwAreA*-*ku70*	Δ*AwPyrG*Δ*Awku70*::*AfpyrG*Δ*AwAreA*::*hygR*	Δ*Awku70*
Δ*AwAreA-lig4*	Δ*AwPyrG*Δ*Awlig4*::*AfpyrG*Δ*AwAreA*::*hygR*	Δ*Awlig4*

### Cultural Conditions

Potato Dextrose Agar (PDA; Becton, Dickinson and Company, Franklin Lakes, NJ, United States) was routinely used to culture *A. westerdijkiae* strains. Minimal media (MM; 6.0 g of NaNO_3_, 0.52 g of KCl, 0.52 g of MgSO_4_⋅7H_2_O, 1.52 g of KH_2_PO_4_, 1 ml of trace elements, 10 g of glucose, and 12.5 g of agar, pH 6.5, in 1 L of distilled water) supported with 15 kinds of nitrogen sources at the concentration of 35 mM were used to study the phenotype of *A. westerdijkiae* and mutants. Nitrogen sources for testing included (NH_4_)_2_HPO_3_, (NH_4_)_2_SO_4_, ammonium citrate, glutamine, sodium glutamate, NaNO_3_, arginine, aspartic acid, cysteine, histidine, methionine, phenylalanine, threonine, tryptophan, and urea. Each experiment was performed three times as biological replicates.

### Fungal Transformation

The generation of *A. westerdijkiae* mutants was performed using protoplast genetic transformation as we described previously (Wang et al., 2019). Positive transformants were verified by diagnostic PCR (Wang G. et al., 2016). The real-time genomic PCR analysis was performed to check the copy number of gene knockout cassettes for transformation as described before (Crespo-Sempere et al., 2013). Briefly, genomic DNA from each strain was used to perform real-time PCR analysis. The number of gene copies was calculated based on the value of Ct (Cycle threshold). The gene *ef1a* (elongation factor 1) was used as the single-copy reference gene. Primers used in this study are listed in Supplementary Table 1.

### Deletion of *AwpyrG* Through 5′-Fluoroorotic Acid Treatment

The deletion of *AwpyrG* was similar to the process of fungal transformation. Actually, we identified the appropriate concentration of 1.6 mg/ml for 5-FOA (Solarbio, Beijing, China) in advance. The two DNA fragments, upstream and downstream of *AwpyrG*, were fused as the deletion cassette. 5-FOA (1.6 mg/ml), uridine (0.5 mg/ml, Solarbio, Beijing, China), and uracil (0.5 mg/ml, Solarbio, Beijing, China) were added to the selection media. The surviving colonies were selected and examined by diagnosed PCR.

### Phenotypic Studies

To assess the growth of *A. westerdijkiae*, 1 μl of conidia suspension (10^6^ conidia/ml) was inoculated into the plate and incubated at 28°C under dark conditions. The growth rate was evaluated by checking the diameter of each colony. For the conidiation study, each plate was washed with 0.1% Tween-80 solution, and the number of conidia was calculated under a microscope with a hemocytometer.

### Ochratoxin A Production Analysis

To investigate the OTA production, six agar plugs (1-cm diameter) from equivalent zones of the fungal surface of the plate were collected and extracted with 2 ml of methanol. After ultrasonic vibrating (30 min) and vortexing (3 min), the supernatant solution was filtered through a 0.22-μm filter into a vial. An Agilent high-performance liquid chromatography (HPLC) system (Agilent Technologies, Santa Clara, CA, United States) was used to analyze OTA concentration, with a mobile phase of acetonitrile/water/acetic acid (99:99:2, v/v).

### Fungal Infection Assays

Fresh pears (*Pyrus bretschneideri* Rehd), grapes (*Vitis vinifera* L.), cheese, and salted meat were selected as food substrates to test the pathogenicity of wild type (WT) and Δ*AwAreA*. The surface of food substrates was disinfected three times with 0.1% NaClO for 10 s and rinsed with sterilized water for 30 s. For pears and grapes, the surface was punctured by a sterilized needle to make a wound with a diameter of 2 mm for inoculation, and 2 μl of conidia suspension (10^6^ conidia/ml) was injected into the wound. For cheese and salted meat, 2 μl of conidia suspension (10^6^ conidia/ml) was inoculated on the surface directly. Inoculation of sterilized water served as control. Each treatment was incubated at 28°C under dark conditions.

### Statistical Analysis

The values were presented as means and standard deviations of three biological replicates. All data were analyzed with IBM SPSS statistics version 20. The mean values were compared by the least significant difference (LSD) and Duncan’s test. It was considered statistically significant when *p* < 0.01.

## Results

### *Aspergillus westerdijkiae* Genome Encodes the Homologs of pyrG and Non-homologous End-Joining Proteins

**FIGURE 1:**
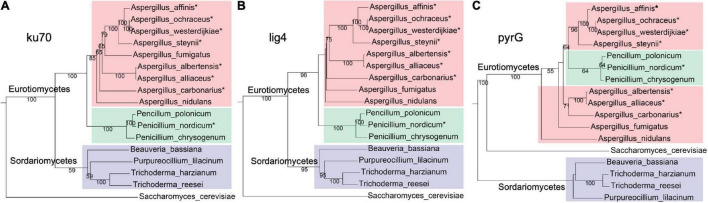
Phylogenetic relationship homologs ku70 **(A)** and lig4 **(B)** and pyrG **(C)**, from fungi. The ochratoxin A (OTA)-producing fungi were marked with an asterisk. Red, green, and purple represent fungi from genus *Aspergillus*, genus *Penicillium*, and class *Sordariomycetes*, respectively. The protein sequences used in this analysis are listed in Supplementary Table 2.

To identify whether pyrG and NHEJ proteins (ku70 and lig4) are present in *A. westerdijkiae* and other ochratoxigenic fungi, BlastP searches were performed using pyrG, ku70, and lig4 from *A. nidulans* as probes, toward several fungal genomes (Supplementary Table 2). As shown in Figure 1, pyrG, ku70, and lig4 from *A. westerdijkiae* were the most related to that from *A. ochraceus*. The evolutionary relationship of ku70 and lig4 (Figures 1A,B) was in accordance with the phylogeny at species levels, with *S. cerevisiae* as an outgroup. However, we could observe a contradiction between the phylogeny of pyrG and the species relationship (Figure 1C). The homologs of pyrG from *Penicillium* clustered with the *A. westerdijkiae* group, while homologs in *Sordariomycetes* seemed more distant to *Eurotiomycetes* compared with outgroup *S. cerevisiae*. These results indicated that NHEJ proteins were conserved in fungi, and pyrG exhibited more diversity in the course of evolution.

### Deletion of *AwpyrG* Leads to Uridine Auxotrophy and 5′-Fluoroorotic Acid Insensitivity of *Aspergillus westerdijkiae*

**FIGURE 2:**
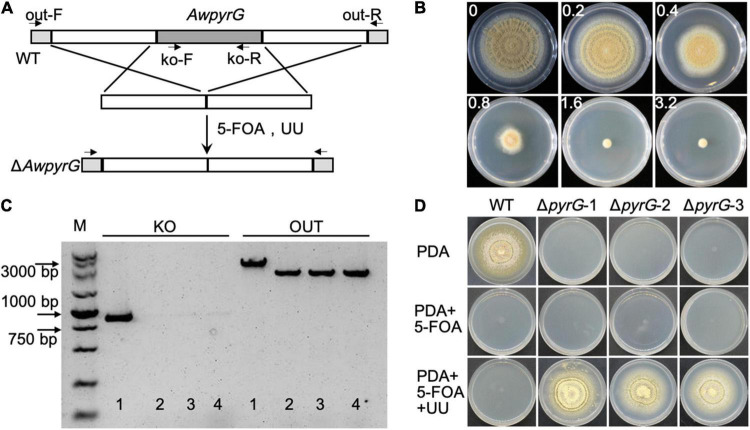
Deletion of *AwpyrG* in *Aspergillus westerdijkiae*. **(A)** Strategy for *AwpyrG* deletion. **(B)** Fungal sensitivity test of *A. westerdijkiae* against 5-FOA. **(C)** PCR identification results of WT (1) and Δ*AwpyrG* (2–4). The fragment KO was amplified with primer pair ko-F/ko-R, and the fragments OUT were amplified with primer pair out-F/out-R. **(D)** The growth status of WT and Δ*AwpyrG* on PDA media with/without 5-FOA and UU for 8 days.

To generate uridine auxotrophic mutants, the *AwpyrG* deletion cassette (combining upstream and downstream of *AwpyrG*) was constructed by the fusion PCR method, followed by transforming to the protoplast of *A. westerdijkiae* (Figure 2A). The positive transformants would be selected by cultural medium containing UU (uridine and uracil) and 5-FOA. We got the appropriate concentration of 1.6 mg/ml for 5-FOA in advance (Figure 2B). As a result, six transformants were obtained. As shown in Figure 2C, the genomic PCR confirmed that the *AwpyrG* gene was deleted in Δ*AwpyrG*. Notably, Δ*AwpyrG* exhibited insensitivity toward 5-FOA with the supplement of UU in PDA medium, while the growth of WT was inhibited (Figure 2D). These results suggested uridine auxotrophic strain was obtained, which could be used for further research.

### The *AfpyrG* Functions as a Selection Marker in the Disruption Non-homologous End-Joining Pathway

To test the availability of uridine auxotrophy selection marker, and to improve the efficiency of gene targeting in *A. westerdijkiae*, genes *Awku70* and *Awlig4* were designed to homologous replace with exogenous *AfpyrG* (Supplementary Figure 2), which were often used as selection markers from *A. fumigatus* (Liu et al., 2018). Successful knockout of *Awku70* and *Awlig4* was identified by PCR diagnosis, and the positive transformants grew well without the supplement of UU. The homologous knockout rates for *Awku70* and *Awlig4* were 6.7 and 11.5%, respectively. There was no significant difference in growth rate and conidia production among WT, Δ*Awku70*, and Δ*Awlig4*, while Δ*AwpyrG* demonstrated the same phenotype in the presence of exogenous UU (Supplementary Figure 3). These data showed exogenous *AfpyrG* functioned as an auxotrophic marker in the transformation of *A. westerdijkiae*.

### Targeting Knockout of *AwAreA* Demonstrates High Efficiency of Homologous Recombination and Low Efficiency of Non-homologous Integration

**FIGURE 3:**
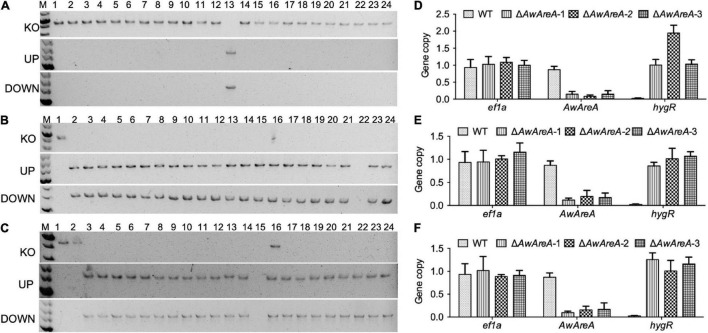
Knockout of *AwAreA* in WT, Δ*Awku70*, and Δ*Awlig4*. PCR identification of Δ*AwAreA* generated from WT **(A)**, Δ*Awku70* **(B)**, and Δ*Awlig4* **(C)**. For each parental strain, a total of 23 transformants (2–24) were randomly selected for PCR identification, with a parental strain (1) as control. The copy number *hygR* cassettes for WT **(D)**, Δ*Awku70* **(E)**, and Δ*Awlig4* **(F)** were determined by real-time genomic PCR analyses.

In order to determine the availability of Δ*Awku70* and Δ*Awlig4*, the NCR regulator *AwAreA* was deleted in WT, Δ*Awku70*, and Δ*Awlig4*. The strategy of mutant generation was similar to Supplementary Figure 2A, except hygromycin B resistant gene (*hygR*) was inserted in the deletion cassettes as a selection marker. A total of 23 transformants that exhibited resistance to hygromycin B were randomly selected for PCR identification. Finally, only one transformant was verified to be positive, for which the amplification of gene *AwAreA* (KO) was absent, and the amplification of UP and DOWN fragments were both positive, indicating the *hygR* cassette integrated at the target loci (Figure 3A). We speculated the *hygR* cassettes inserted randomly into chromosomes in other transformants mediated by NHEJ pathway. The efficiency of homologous replacement was 4.3%, similar to the efficiency of 7.8% for the knockout of *AocreA* (Wang et al., 2020). For the parental strain Δ*Awku70*, a total of 23 generated Δ*AwAreA* were obtained, with the homologous replacement efficiency of 95.7% (Figure 3B). For the parental strain Δ*Awlig4*, the homologous replacement efficiency was 87.0% (Figure 3C). These results indicated the efficiency of homologous replacement increased dramatically by the disruption of *Awku70* and *Awlig4*.

In addition to the low efficiency of gene knockout, another worry for transformation is that ectopic insertions accompanied with homologous replacement affect the function identification of target genes. Real-time genomic PCR analyses were performed to determine the copy numbers of *hygR* cassettes that were integrated into the genomes of Δ*AwAreA* generated from different strains. As shown in Figure 3D, the WT and the generated Δ*AwAreA*-WT (three deletion mutants) had one copy of reference gene *ef1a*. The WT strain possessed one copy of *AwAreA*, while the three mutants had lost the gene. The *hygR* cassette could not be detected in the WT strain. Two mutants possessed one copy of *hygR*, while one of the mutants had two copies of *hygR*, which indicated a *hygR* cassette happened during ectopic insertion in the genome. For Δ*Awku70* and Δ*Awlig4*, all of the generated Δ*AwAreA* strains had one copy of *hygR* (Figures 3E,F). These data demonstrated that deletion of *Awku70* and *Awlig4* led to the low efficiency of non-homologous integration in *A. westerdijkiae*.

### The Growth and Conidiation of *Aspergillus westerdijkiae* Are Affected by Nitrogen Sources and Modulated by *AwAreA*

**FIGURE 4:**
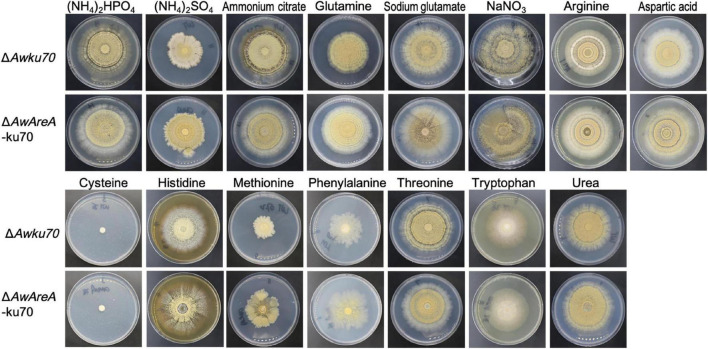
Colony view of *Aspergillus westerdijkiae*Δ*Awku70* and Δ*AwAreA*-ku70 grown on media containing different nitrogen sources.

**FIGURE 5:**
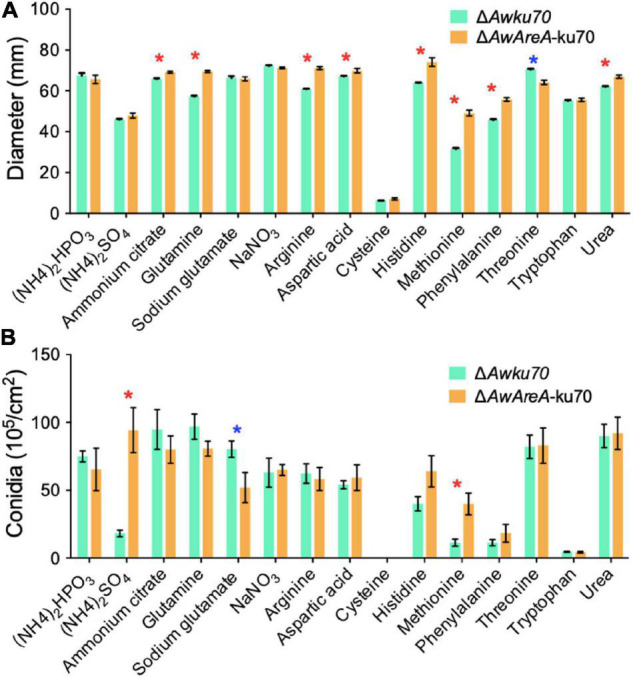
Effect of *AwAreA* deletion on the colony growth and conidiation of *Aspergillus westerdijkiae* on different nitrogen sources. **(A)** Diameter of Δ*Awku70* and Δ*AwAreA*-ku70. **(B)** The conidia number of Δ*Awku70* and Δ*AwAreA*-ku70. The asterisk in red/blue color indicates a significant increase/decrease between the corresponding values (*p* < 0.01) with three biological replicates.

The function of *AwAreA* was further studied with mutant Δ*AwAreA*-ku70 under the comparison of Δ*Awku70*. A series of differences related to colony morphology for *A. westerdijkiae* were observed when cultivated with media supplemented with different nitrogen sources. As shown in Figure 4, all the nitrogen sources for testing could be utilized for growth except cysteine. The growth rate of Δ*Awku70* supplemented with methionine, (NH_4_)_2_SO_4_, and phenylalanine was obviously slower than that of others, and the colony edge showed an irregular shape. We could also observe the color alterations and different patterns of haloes in colonies when Δ*Awku70* was cultivated with different nitrogen sources. For example, the colony was yellow on glutamine medium while white on tryptophan medium, and a white halo was produced around the colony on arginine medium. These morphological characteristics seemed to be correlated with the production of conidiospore, pigments, and other secondary metabolites (Visagie et al., 2014). *AwAreA* regulated the growth of *A. westerdijkiae* on most of the nitrogen for testing. Deletion of *AwAreA* led to the decrease of growth rate on the media supplemented with threonine. A significant increase in the growth caused by *AwAreA* deletion could be observed on the media supplemented with ammonium citrate, glutamine, arginine, aspartic acid, histidine, phenylalanine, and urea (Figure 5A). Meanwhile, the number of conidia dramatically increased on (NH_4_)_2_SO_4_ and methionine media but decreased on sodium glutamate medium (Figure 5B). The results demonstrated that *AwAreA* regulated the growth and conidiation of *A. westerdijkiae* in response to nitrogen sources.

### *AwAreA* Regulates the Biosynthesis of Ochratoxin A Responding to Nitrogen Sources

**FIGURE 6:**
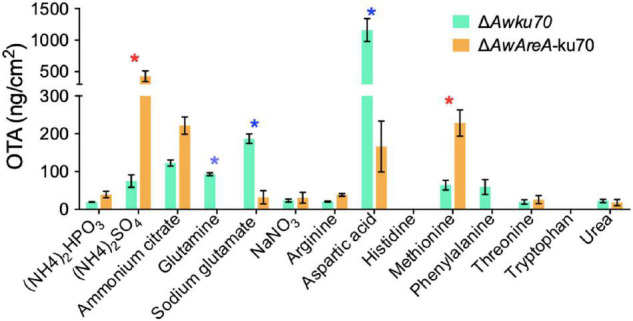
Ochratoxin A (OTA) production of Δ*Awku70* and Δ*AwAreA-*ku70 on media containing different nitrogen sources. The asterisk in red/blue color indicates a significant increase/decrease between the corresponding values (*p* < 0.01) with three biological replicates.

OTA is the most important and toxic secondary metabolite produced by *A. westerdijkiae*. Here, we found by HPLC assay that OTA production was affected by nitrogen sources. As shown in Figure 6, the concentration of OTA reached 1,000 ng/cm^2^ of Δ*Awku70* on the media containing aspartic acid as a nitrogen source, while tryptophan could not contribute to the biosynthesis of OTA. For other nitrogen, the concentration of OTA of Δ*Awku70* was in the range of 19.4–186.9 ng/cm^2^. Intriguingly, the OTA production influenced by the deletion of *AwAreA* was different based on nitrogen sources. For (NH_4_)_2_SO_4_ and methionine, the concentration of OTA dramatically increased after *AwAreA* deletion. The production of OTA significantly decreased in the case of glutamine and aspartic acid as nitrogen sources. Especially, the biosynthesis of OTA was completely suppressed in Δ*AwAreA*-ku70 for glutamine. These results indicated that the biosynthesis of OTA was affected by nitrogen sources and modulated by gene *AwAreA*.

### *AwAreA* Plays Different Roles in Pathogenicity According to Food Substrates

**FIGURE 7:**
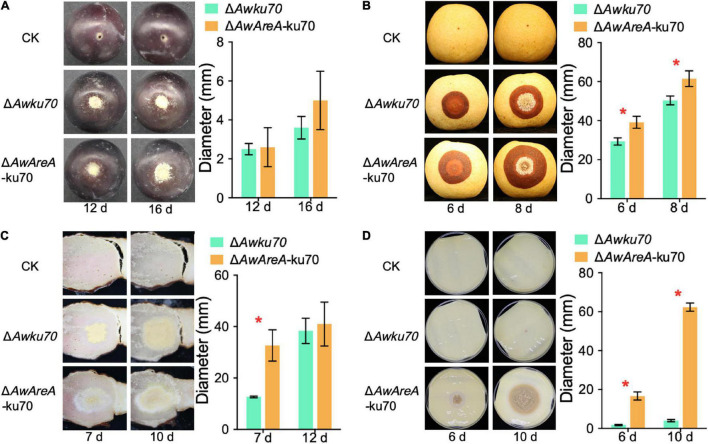
Pathogenicity assay of Δ*Awku70* and Δ*AwAreA*-ku70 of *Aspergillus westerdijkiae* on grape **(A)**, pear **(B)**, salted meat **(C)**, and cheese **(D)**. The red asterisk in the histogram indicates a significant difference between the corresponding values (*p* < 0.01) with three biological replicates.

OTA-producing fungi are known to colonize food substrates such as fruits, salted meat, and cheese. We investigated the influences of *AwAreA* deletion on the pathogenicity of *A. westerdijkiae* toward pear, grape, salted meat, and cheese. The assay revealed that the loss of *AwAreA* had varying degrees of effect on fungal infection. As shown in Figure 7A, *A. westerdijkiae* had infected and formed a colony on grapes at 12 and 16 days after inoculation. There was no significant difference between Δ*Awku70* and Δ*AwAreA*-ku70. On pear infection, the lesion of Δ*AwAreA*-ku70 demonstrated an increase compared with Δ*Awku70*, indicating that *AwAreA* is a negative regulator in *A. westerdijkiae* for pear infection (Figure 7B). For salted meat, we could observe an increase in colony diameter and a change in colony color after 7 days of inoculation of *A. westerdijkiae* due to *AwAreA* deletion. The color of the colony, which changed from yellow to white, resulted from the decrease of yellow conidia. Ten days after inoculation, the diameter of Δ*Awku70* and Δ*AwAreA*-ku70 tended to be the same (Figure 7C). As shown in Figure 7D, Δ*Awku70* could hardly grow on cheese after 6 and 10 days after inoculation but survived after *AwAreA* deletion. In order to figure out the influence of the NHEJ pathway toward fungal virulence, we compared the pathogenicity of WT, Δ*Awku70*, and Δ*Awlig4* on pears (Supplementary Figure 4). There was no difference among the lesions at 6 and 8 days after inoculation. These results proved the practicality of the genetically engineered strains and indicated the negative regulatory role of *AwAreA*.

## Discussion

The limited availability of selection markers in transformation represents a bottleneck for genetic studies in *A. westerdijkiae* and other OTA-producing fungi. Researchers are still genetically inspecting *A. westerdijkiae* for its control and application in agriculture, food, and industry. Based on the biosynthetic pathway of uridine, we reported the establishment of uridine auxotrophic mutant Δ*AwpyrG* of *A. westerdijkiae*, *via* a positive selection of 5-FOA. An alternative strategy to obtain uridine auxotrophic mutants is to utilize chemical or UV mutagenesis. We selected a replacement strategy rather than mutagenesis to isolate the desired auxotrophic strains, for the avoidance of complications and the undetected mutations arising from the non-specific manner. Compared with antibiotic-selective markers such as hygromycin and geneticin/G418, the auxotrophic-selective marker demonstrated higher efficiency with fewer false-positive transformants. Another advantage for the use of auxotrophy in transformation is that no exogenous antibiotic-resistant genes would be introduced. Furthermore, recycling the marker gene *pyrG via* the method in this study enables us to use the selective marker repeatedly when multiple genetic manipulations would be performed on a single strain.

The low-efficiency gene targeting system in ochratoxigenic fungi hindered the in-depth research regarding the occurrence and development of OTA and their producing fungi, the molecular biology study of which was limited to gene function identification by gene homologous knockout. However, a series of technologies such as protein pull down and green fluorescent protein (GFP) fusion based on high efficiency of gene target have been reported in *A. flavus* and *A. nidulans* (Ribeiro Liliane et al., 2019; Yang et al., 2021). Here, we constructed two mutants Δ*Awku70* and Δ*Awlig4* by the disruption of the NHEJ pathway. Δ*Awku70* and Δ*Awlig4* dramatically increased the gene targeting efficiency to 95.7 and 87.0%, while the efficiency of WT was 4.3% in disruption *AwAreA*. The isolates supported us to obtain gene knockout transformants easily and to perform deeper research based on homologous integration. It was interesting that deletion of *ku70* and *lig4* has not enhanced the efficiency of the homologous target in some filamentous fungi (Liu et al., 2018). Until now, all of the ochratoxigenic fungi are distributed in the genera *Aspergillus* and *Penicillium*. Given the conservative NHEJ in OTA-producing fungi (Figure 1), we speculated that this strategy could be used as a reference in other ochratoxigenic fungi.

Furthermore, the growth rate and conidia number of Δ*AwpyrG*, Δ*Awku70*, and Δ*Awlig4* were evaluated. These data showed the strains could be used to study the regulatory function of genes toward the development of *A. westerdijkiae*. The virulence Δ*Awku70* and Δ*Awlig4* in pears enabled them to be used in exploring the interaction between plants and fungi.

The function of *AwAreA*, the major regulator of nitrogen utilization, was characterized in this study. Similar phenotypes were demonstrated among the Δ*AwAreA* mutants generated from WT, Δ*Awku70*, and Δ*Awlig4*. This demonstrated that Δ*Awku70* and Δ*Awlig4* could be used to study gene function in the future. Therefore, the subsequence studies of *AwAreA* were performed in a *ku70* deletion background strain. *A. westerdijkiae* showed different abilities in the utilization of different nitrogen for growth, conidiation, and OTA biosynthesis. *AwAreA* deletion led to better utilization of ammonium citrate, glutamine, arginine, etc., for growth but worse utilization of threonine by examination of the colony diameter. The ability of *A. westerdijkiae* to utilize nitrogen for conidiation was also affected by *AwAreA* deletion. A recent study reported that the number of conidia significantly decreased by disruption *AreA* in *A. flavus* when cultivated in a nutritious yeast extract sucrose medium (Fasoyin et al., 2019). Taken together, it is reasonable to speculate that *AreA* plays different roles for various nitrogen.

The biosynthesis of mycotoxins is influenced by nitrogen availability and nitrogen regulators. *AreA* is a positive regulator in the production of aflatoxins, zearalenone, and fumonisin (Ehrlich et al., 2011; Fasoyin et al., 2019). The GATA transcription factor *AreA* has binding sites in the promoters of key genes in the secondary metabolite biosynthetic cluster. The deletion of *AwAreA* caused a significant reduction of OTA production when *A. westerdijkiae* was cultivated on the media containing glutamine, sodium glutamate, and aspartic acid, indicating the role as a positive regulator. However, the production of OTA increased due to the deletion of *AwAreA* when *A. westerdijkiae* was cultivated in (NH_4_)_2_SO_4_ and methionine media. *AwAreA* may employ a complicated mechanism in the regulation of OTA biosynthesis accompanied by the change of nitrogen sources.

Fruit, cheese, and salted meat are widely reported to be infected by ochratoxigenic fungi. It is well known that the type and quantity of nitrogen differ among diverse food substrates. Fruits contain various nitrogenous compounds, including ammonium cations and organic nitrogenous compounds. The existence of bioactive peptides in cheese and abundant protein in salted meat has been widely described as nitrogen sources (Parra-Ocampo et al., 2020), and salted meat is also rich in protein (Huang et al., 2014). Combining the results analyzed *in vitro*, it is reasonable that *AwAreA* plays various roles in the infection of food substrates containing different nitrogen sources. In *A. flavus*, Δ*AreA* was significantly impaired in conidiation compared to the WT (Fasoyin et al., 2019). This is consistent with the previous results that were revealed by *A. westerdijkiae* in salted meat, while on cheese, *A. westerdijkiae* was viable only if there is loss of function of *AwAreA*. Taken together, it is speculated that *AwAreA* acts as a negative regulator in the infection process of *A. westerdijkiae*.

## Conclusion

*A. westerdijkiae* is the main contributor of OTA contamination in food. The uridine auxotrophy was obtained by the deletion of *AwpyrG*, indicating that the first auxotrophic marker is available in *A. westerdijkiae*. With further development regarding the efficiency of gene target, we constructed two mutants that lack *Awku70* and *Awlig4* in the NHEJ pathway, demonstrating the high efficiency of HR and low efficiency of non-homologous integration. The growth, conidiation, and pathogenicity were proved to be consistent among WT, Δ*Awku70*, and Δ*Awlig4*. Furthermore, the function of NCR regulator *AwAreA* was identified with *AwAreA* deletion mutant Δ*AwAreA*-ku70 and the control strain Δ*Awku70*. The growth, conidiation, and OTA production were regulated by nitrogen sources and modulated by *AwAreA*. Pathogenicity assays showed that *AwAreA* acted as a negative regulator in the infection of food substrates. The genetic methods and engineered strains enabled to use *A. westerdijkiae* in the study of OTA biosynthesis and regulation. Furthermore, the function of *AwAreA* was identified, which provides theoretical insights into the prevention of OTA-producing fungi.

## Data Availability Statement

The datasets presented in this study can be found in online repositories. The names of the repository/repositories and accession number(s) can be found below: https://www.ncbi.nlm.nih.gov/genbank/, OM283273.

## Author Contributions

GW and FX designed the experiment. GW, YL, BY, WW, and PS performed the experiments. EL and GW analyzed the data. GW wrote the manuscript. FX revised the manuscript. All authors contributed to the article and approved the submitted version.

## Conflict of Interest

The authors declare that the research was conducted in the absence of any commercial or financial relationships that could be construed as a potential conflict of interest.

## Publisher’s Note

All claims expressed in this article are solely those of the authors and do not necessarily represent those of their affiliated organizations, or those of the publisher, the editors and the reviewers. Any product that may be evaluated in this article, or claim that may be made by its manufacturer, is not guaranteed or endorsed by the publisher.
