# Supplementary material for: AwAreA Regulates Morphological Development, Ochratoxin A Production, and Fungal Pathogenicity of Food Spoilage Fungus Aspergillus westerdijkiae Revealed by an Efficient Gene Targeting System
Source: Front Microbiol. 2022 Mar 31;13:857726. doi: 10.3389/fmicb.2022.857726 (PMC9009206; doi:10.3389/fmicb.2022.857726)
Supplement: Supplementary file 1 [file Data_Sheet_1.docx]

Supplementary Material

# Supplementary Figures


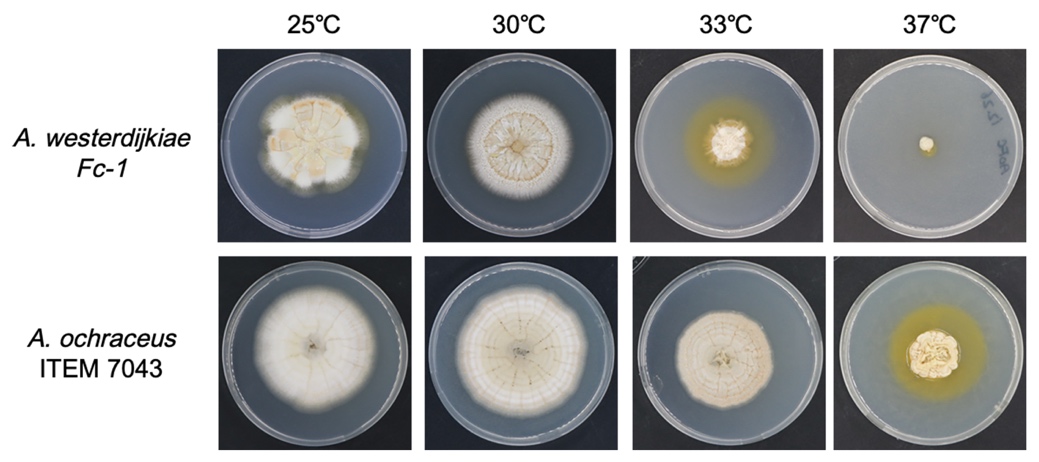


**Figure S1.** The morphological difference of *A. westerdijkiae* and *A. ochraceus* cultured on Czapek Yeast Autolysate agar.


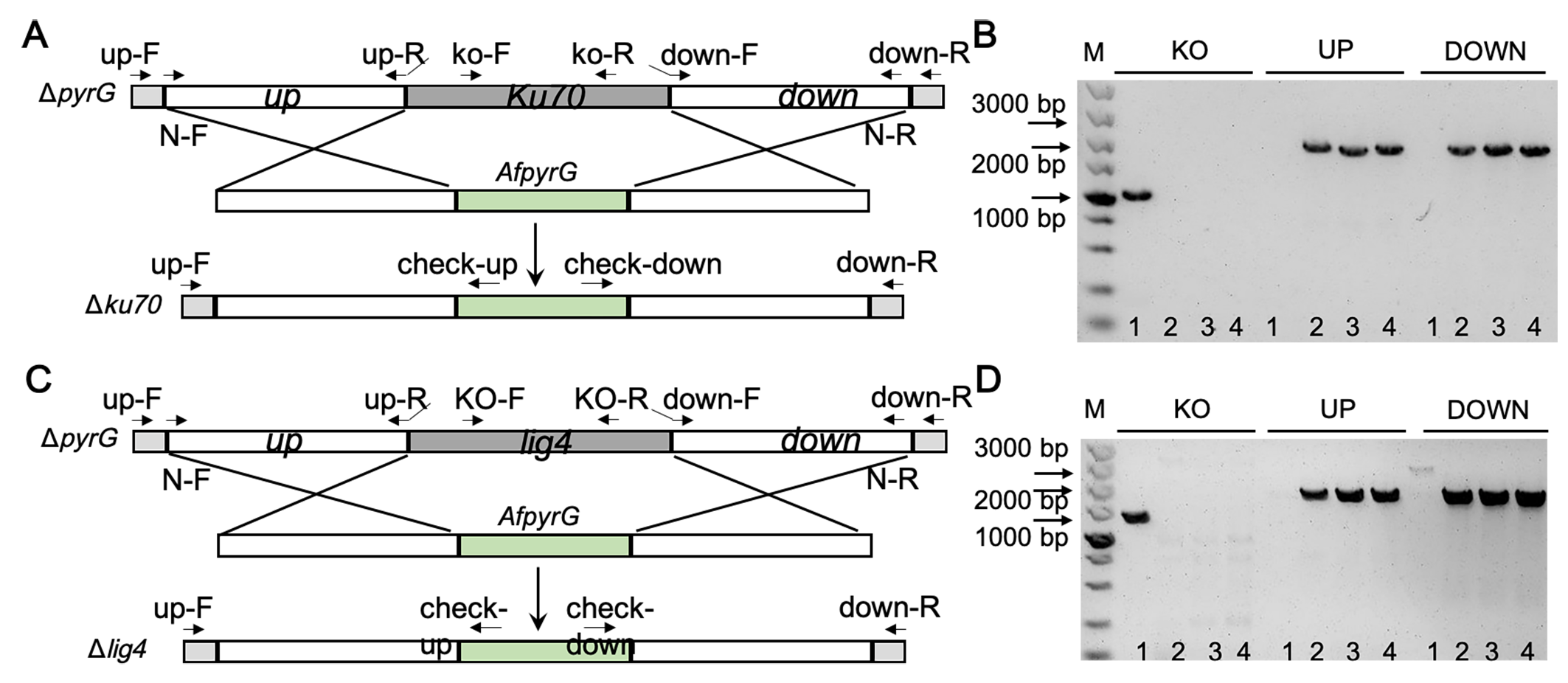


**Figure S2.** Deletion of *Awku70* and *Awlig4* in *A. westerdijkiae*. (A) Strategy for *Awku70* deletion; (B) PCR identification of Δ*Awku70* mutants. Each primer pairs corresponds to one WT (1) and three Δ*Awku70* mutants (2, 3 and 4). The fragment KO was amplified with primer pair ko-F/ko-R, the fragments OUT were amplified with primer pair up-F/check-up, and the fragments DOWN were amplified with primer pair down-R/check-down; (C) Strategy for *Awlig4* deletion; (D) PCR identification of Δ*Awlig4* mutants.


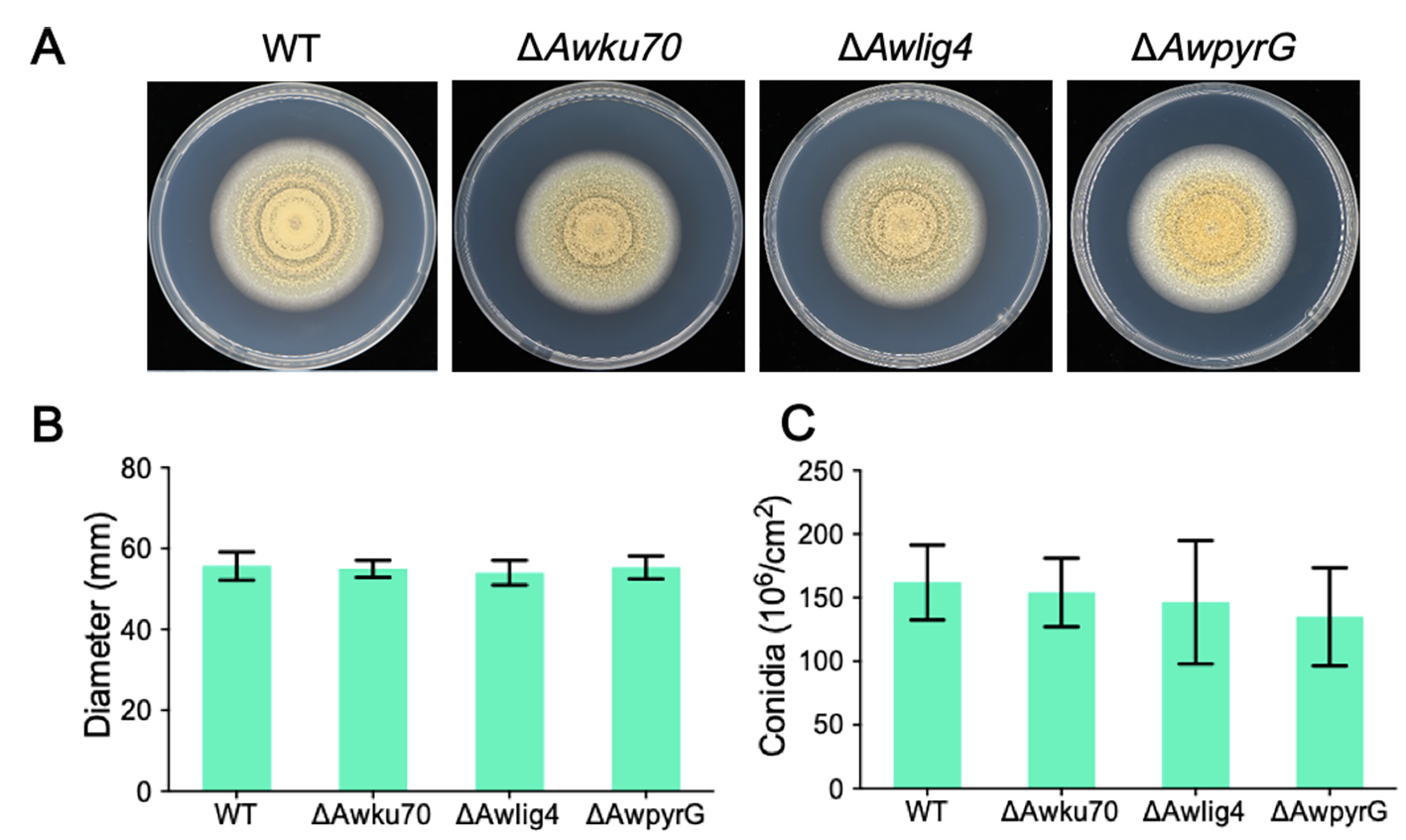


**Figure S3.** The growth and conidiation of WT, Δ*Awku70*, Δ*Awlig4* and Δ*AwpyrG*. (A) Colony view of the WT and mutants of *A. westerdijkiae* at 5 days after inoculation; (B) The colony diameter of WT and mutants; (C) Conidia production of WT and mutants.


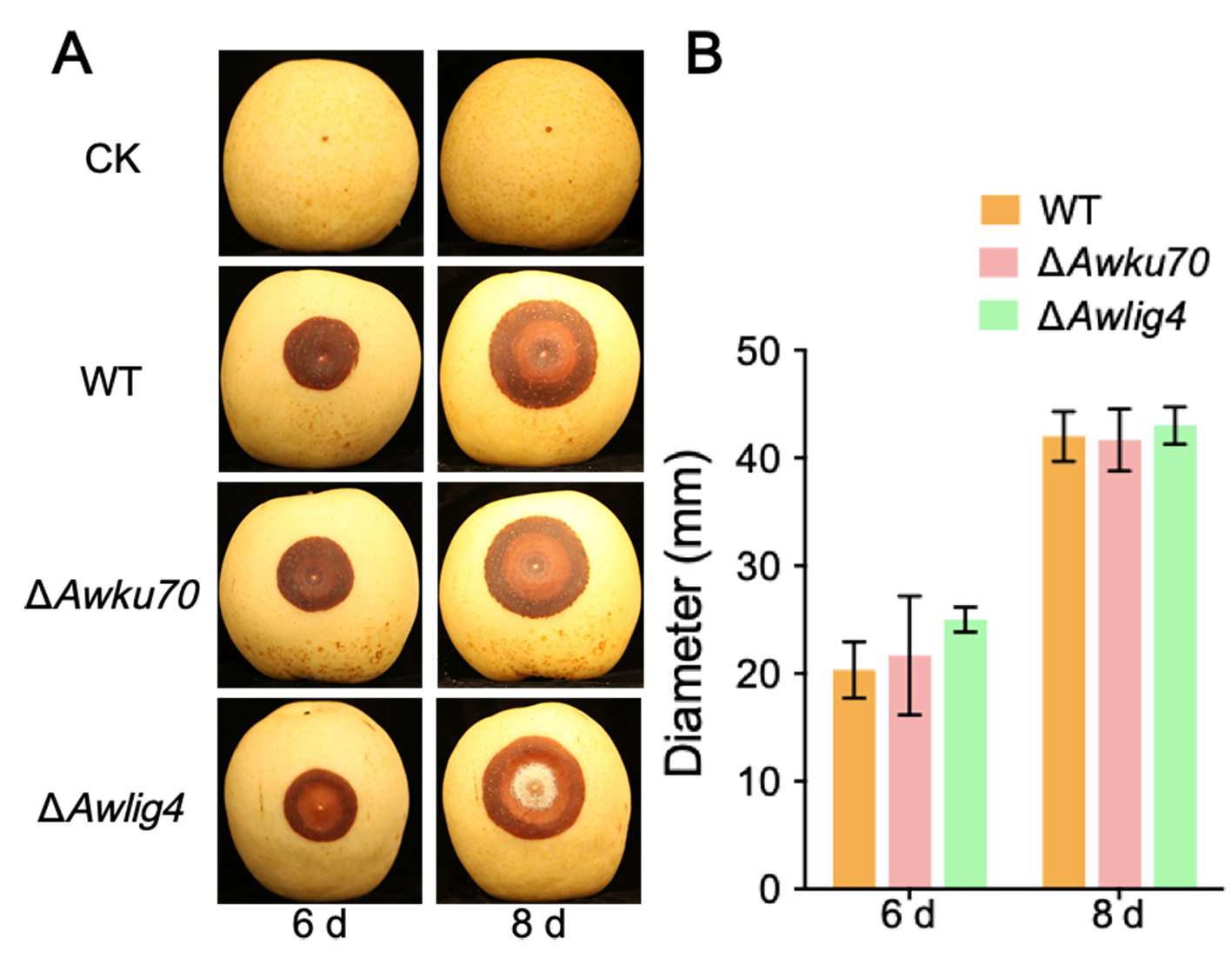


**Figure S4.** Pathogenicity assay for WT, Δ*Awku70* and Δ*Awlig4* on pears. (A) Pears infected incubated at 28°C for six and nine days and photographed. (B) The scab diameters of pears.

# Supplementary Tables

**Table S1.** Primers used in this study.

| **Primers** | **Oligonucleotide sequence (5’-3’)** | **Uses** |
| --- | --- | --- |
| pyrG-up-F | cgcccactcgaacttcctag | *AwpyrG* deletion cassette |
| pyrG-up-R | ttctgcaacacggctgttatctctcgaaccgtcgatatcggatggagggg |  |
| pyrG-down-F | ccccgcattacccctccatccgatatcgacggttcgagagataacagccg |  |
| pyrG-down-R | ggcatcatatgccagtcgatg |  |
| N-pyrG-F | gccatgttcttcatcctggc |  |
| N-pyrG-R | gcgtctaacgtgatcgattcc |  |
| pyrG-KO-F | gcaattgacatactccgagcgg | Identification of *AwpyrG* deletion mutants |
| pyrG-KO-R | cacccctcctcctgataacg |  |
| pyrG-OUT-F | cgcccactcgaacttcctag |  |
| pyrG-OUT-R | ggcatcatatgccagtcgatg |  |
| AfpyrG-F | gagagttattctgtgtctga | *AfpyrG* amplification |
| AfpyrG-R | attctgtctgagaggaggc |  |
| ku70-up-F | ccgttaggtggccgtcttag | *Awku70* deletion cassette |
| ku70-up-R | acatatttcgtcagacacagaataactctcggcaagggataaggaatggacc |  |
| ku70-down-F | cacgcatcagtgcctcctctcagacagaatcgccatgatgaatctgcgag |  |
| ku70-down-R | ggtggttcgcctcttccatc |  |
| N-ku70-F | aggatacttgccctcacagc |  |
| N-ku70-R | cggcaccaggacaatgtctg |  |
| ku70-KO-F | ccttcttgggtgcatgtccac | Identification of *Awku70* deletion mutants |
| ku70-KO-R | agccagccaggaactcctac |  |
| ku70-up-F | ccgttaggtggccgtcttag |  |
| check-up | gtcaggtacagctagaatgggg |  |
| check-down | gtccctcaggacaagtcgacc |  |
| ku70-down-R | ggtggttcgcctcttccatc |  |
| lig4-up-F | cctagctccccttccctcac | *Awlig4* deletion cassette |
| lig4-up-R | acatatttcgtcagacacagaataactctcgttactcctggacaacaggcg |  |
| lig4-down-F | cacgcatcagtgcctcctctcagacagaatggaatatccacggcaaacccc |  |
| lig4-down-R | cctagcgagactcccgagtc |  |
| N-lig4-F | gggtatgtacattgccctggtg |  |
| N-lig4-R | cttggtcgagcagcgatcac |  |
| lig4-KO-F | gacgatcgaggaggtcaacg | Identification of *Awlig4* deletion mutants |
| lig4-KO-R | catattgagcatcaccacccg |  |
| lig4-up-F | cctagctccccttccctcac |  |
| check-up | gtcaggtacagctagaatgggg |  |
| check-down | gtccctcaggacaagtcgacc |  |
| lig4-down-R | cctagcgagactcccgagtc |  |
| hygR-F | ggaggtcaacacatcaatgcc | *hygR* amplification |
| hygR-R | ctactctattcctttgccctcgg |  |
| AreA-up-F | cacgctaaggtggacagtcc | *AwAreA* deletion cassette |
| AreA-up-R | caaaataggcattgatgtgttgacctccgaatcgggcggactaagagg |  |
| AreA-down-F | ctcgtccgagggcaaaggaatagagtaggacgagaccgaagacagca |  |
| AreA-down-R | gaggtgggagagggagtcac |  |
| N-AreA-F | agtcctaactacggagcggt |  |
| N-AreA-R | gctggataggagggataggga |  |
| AreA-KO-F | gcaagatccgttagccaccc | Identification of *AwAreA* deletion mutants |
| AreA-KO-R | ggagagatcgggatcgtggg |  |
| AreA-up-F | cacgctaaggtggacagtcc |  |
| check-up | ggctgatctgaccagttgcc |  |
| check-down | ggctgtgtagaagtactcgcc |  |
| AreA-down-R | gaggtgggagagggagtcac |  |
| ef1a-q-F | gtctggtgatgctgccatcg | Detecting the copy number of genes |
| ef1a-q-R | ccttgatgacaccgacagcg |  |
| AwAreA-q-F | gttcttcggcttgcagttgcc |  |
| AwAreA-q-R | cgatacatgatggctaccgcg |  |
| hygR-q-F | gtcacgttgcaagacctgcc |  |
| hygR-q-R | cgcgcatatgaaatcacgcc |  |

**Table S2.** The information of fungal genomes and protein sequences used for phylogenetic analysis.

| Species | Strain name | Database | Gene ID/Accession No. | | |
| --- | --- | --- | --- | --- | --- |
|  |  |  | pyrG | ku70 | lig4 |
| *Aspergillus westerdijkiae* | Fc-1 | NCBI | AoFC_11450 | AoFC_02106 | AoFC_11278 |
| *Aspergillus ochraceus* | ITEM 7043 | JGI | 123771 | 713333 | 115905 |
| *Aspergillus affinis* | CBS 129190 | JGI | 550131 | 562692 | 539609 |
| *Aspergillus steynii* | IBT 23096 | NCBI | PLB47093.1 | PLB53640.1 | PLB47303.1 |
| *Aspergillus fumigatus* | Af293 | NCBI | EAL93132.1 | EAL91824.1 | EAL91408.1 |
| *Aspergillus albertensis* | IBT 14317 | JGI | 149811 | 47482 | 150391 |
| *Aspergillus alliaceus* | CBS 536.65 | JGI | 301461 | 268778 | 309473 |
| *Aspergillus carbonarius* | 5010 | JGI | 210262 | 209975 | 206313 |
| *Aspergillus nidulans* | FGSC_A4 | AspGD | AN6157 | AN7753 | AN0097 |
| *Pencillum polonicum* | IBT 4502 | NCBI | OQD71243.1 | OQD66141.1 | OQD64158.1 |
| *Penicillium nordicum* | DAOMC 185683 | NCBI | KOS45171.1 | KOS40310.1 | KOS48797.1 |
| *Penicillium chrysogenum* | P2niaD18 | NCBI | KZN92690.1 | KZN91043.1 | KZN88464.1 |
| *Beauveria bassiana* | ARSEF 2860 | NCBI | EJP65537.1 | EJP64188.1 | EJP61643.1 |
| *Purpureocillium lilacinum* | PLBJ-1 | NCBI | VFPBJ_05735 | VFPBJ_00677 | VFPBJ_02503 |
| *Trichoderma harzianum* | CBS 226.95 | NCBI | PTB56790.1 | PTB61214.1 | PTB49448.1 |
| *Trichoderma reesei* | QM6a | NCBI | EGR52858.1 | EGR47890.1 | EGR50407.1 |
| *Saccharomyces cerevisiae* | S288C | NCBI | DAA07631.1 | DAA10185.1 | DAA10787.1 |
